# ST6Gal-I expression in ovarian cancer cells promotes an invasive phenotype by altering integrin glycosylation and function

**DOI:** 10.1186/1757-2215-1-3

**Published:** 2008-10-01

**Authors:** Daniel R Christie, Faheem M Shaikh, John A Lucas, John A Lucas, Susan L Bellis

**Affiliations:** 1 Department of Obstetrics and Gynecology, University of Alabama at Birmingham, Birmingham, AL 35294, USA; 2 Department of Physiology and Biophysics, University of Alabama at Birmingham, Birmingham, AL 35294, USA

## Abstract

**Background:**

Ovarian adenocarcinoma is not generally discovered in patients until there has been widespread intraperitoneal dissemination, which is why ovarian cancer is the deadliest gynecologic malignancy. Though incompletely understood, the mechanism of peritoneal metastasis relies on primary tumor cells being able to detach themselves from the tumor, escape normal apoptotic pathways while free floating, and adhere to, and eventually invade through, the peritoneal surface. Our laboratory has previously shown that the Golgi glycosyltransferase, ST6Gal-I, mediates the hypersialylation of β_1 _integrins in colon adenocarcinoma, which leads to a more metastatic tumor cell phenotype. Interestingly, ST6Gal-I mRNA is known to be upregulated in metastatic ovarian cancer, therefore the goal of the present study was to determine whether ST6Gal-I confers a similarly aggressive phenotype to ovarian tumor cells.

**Methods:**

Three ovarian carcinoma cell lines were screened for ST6Gal-I expression, and two of these, PA-1 and SKOV3, were found to produce ST6Gal-I protein. The third cell line, OV4, lacked endogenous ST6Gal-I. In order to understand the effects of ST6Gal-I on cell behavior, OV4 cells were stably-transduced with ST6Gal-I using a lentiviral vector, and integrin-mediated responses were compared in parental and ST6Gal-I-expressing cells.

**Results:**

Forced expression of ST6Gal-I in OV4 cells, resulting in sialylation of β1 integrins, induced greater cell adhesion to, and migration toward, collagen I. Similarly, ST6Gal-I expressing cells were more invasive through Matrigel.

**Conclusion:**

ST6Gal-I mediated sialylation of β1 integrins in ovarian cancer cells may contribute to peritoneal metastasis by altering tumor cell adhesion and migration through extracellular matrix.

## Background

The α2–6 linkage of sialic acids to *N*-acetyllactosamine structures (Galβ1–4GlcNAc) is a Golgi-mediated process facilitated by the enzyme, β-galactoside α2–6-sialyltransferase (ST6Gal-I). Variant α2–6 sialylation can have a wide array of biologic and pathogenic consequences, including alterations in immune response and embryogenesis, as well as a role in the development and progression of some cancers [1]. There are several recognized substrates upon which ST6Gal-I is known to act: β1 integrin [2], E-selectin, ICAM-1, and VCAM-1 [3]. Perturbation of normal ST6Gal-I functioning fundamentally alters cell behavior by modulating normal cell interactions with the surrounding environment.

The overexpression of ST6Gal-I is well documented in several diverse cancer types. These cancers include: colorectal [4], cervical [5], breast [6], hepatocellular [7], and certain cancers of the head and neck [8]. ST6Gal-I is upregulated by oncogenic ras [9-11] thus accounting for the increased enzyme expression in the various tumor types [2]. Our group has reported that forced expression of ST6Gal-I in SW48 colonocytes, which lack endogenous sialyltransferase activity, caused increased binding to collagen I and laminin, and increased cell motility [12]. This change in cell behavior was shown to be a consequence of the hypersialylation of the β_1 _integrin. Though incompletely understood, β_1 _hypersialylation could modify integrin-dependent cell responses through a change in receptor conformation, by masking functional domains within the integrin heterodimer, by affecting integrin interaction with other membrane bound proteins or glycolipids, or by another, as yet, unrecognized mechanism [2]. Lin and colleagues demonstrated that forced expression of ST6Gal-I in MDA-MB-435 human mammary carcinoma cells resulted in increased adhesion to collagen IV, reduced cell-cell adhesion, and increased capacity for invasion [13]. Conversely, introduction of antisense oligonucleotides to ST6Gal-I in colon cancer cells reduced the cells' ability to form colonies and to invade [14]. Taken in sum, these results suggest that overexpression of ST6Gal-I results in a phenotype consistent with aggressive metastasis. In fact, increased tumor levels of ST6Gal-I have been correlated with poorer patient prognosis [15,6], though there are also reports suggesting that ST6Gal-I activity is not predictive of outcome [16,17].

The role of ST6Gal-I in ovarian carcinoma has not been as clearly defined as its effect in some other tumors, namely colon and breast. Nonetheless, there are recent data indicative of the emerging attention to the importance of sialylation in ovarian cancer. High-throughput techniques have yielded evidence that ST6Gal-I is up-regulated in epithelial ovarian malignancy. For example, proteomic analysis revealed α2–6 sialylation to be proportionally favored over α2–3 sialylation [18]. This mirrors the results of Wang and colleagues who showed increased mRNA levels of ST6Gal-I and decreased levels of the α2–3 sialyltransferase, ST3Gal-VI in ovarian cancer [19]. These enzymes can compete for the linkage of sialic acids to terminal Galβ1–4GlcNAc, and thus the findings indicate that there is preference for α2–6 sialylation in the ovary with malignant transformation. Despite these observed differences in ST6Gal-I mRNA and global cell surface sialylation, a direct examination of ST6Gal-I protein in ovarian tumor cells has not previously been attempted. As well, there is limited information regarding the functional consequences of ST6Gal-I upregulation in ovarian carcinoma. Casey and colleagues treated OVCAR5 ovarian carcinoma cells with neuraminidase enzyme to remove sialic acids and found that this decreased migration toward fibronectin, and reduced invasion through Matrigel [20]. However, the neuraminidase enzyme does not discriminate between α2–6 and α2–3-linked sialic acids, and therefore the changes in cell migration and invasion could not be directly ascribed to ST6Gal-I activity.

In the present study, we screened three separate ovarian carcinoma cell lines for endogenous expression of ST6Gal-I, and found that two of these were positive for ST6Gal-I protein. The third, the OV4 cell line, had negligible levels of the enzyme and therefore, to assess the effects of α2–6 sialylation on promoting the tumor cell phenotype, we forced ST6Gal-I expression and evaluated integrin-dependent cell behaviors. ST6Gal-I expression, with consequent β_1 _integrin hypersialylation, induced increased adhesion to collagen I, migration toward collagen I, and invasiveness through Matrigel. Our results suggest a potential role for variant sialylation in the dissemination of ovarian carcinoma.

## Methods

### Ovarian carcinoma cell lines

The ovarian carcinoma cell line SKOV3 was generously gifted to us by Dr. Janet Price (MD Anderson, Houston, TX), whereas the OV4 cell line was a generous gift from Dr. Timothy Eberlein (Harvard, Cambridge, MA). The PA1 cell line was purchased commercially through ATCC (Manassas, VA). PA1 cells were cultured and grown in Eagle's minimal essential medium (MEM) supplemented with 10% fetal bovine serum (FBS, Hyclone, Logan, UT) and penicillin, streptomycin, and amphotericin B. OV4 and SKOV3 cells were cultured and grown in Dulbecco's modified Eagle's MEM/Ham's F-12 50:50 (DMEM/F12) supplemented with 10% FBS, penicillin, streptomycin, and amphotericin B. Cells were maintained at 37°C in 5% CO_2 _and passaged two to three times per week.

### Western blotting

Cells were lysed in buffer composed of 50 mM Tris-HCl (pH 7.4) containing 1% Triton X-100, and a protease inhibitor cocktail (Roche Applied Bioscience). Protein concentrations of the lysates were determined using a modified Bradford Assay (Sigma, St. Louis, MO). Proteins were resolved by reducing SDS-PAGE, and transferred to polyvinylidene difluoride membranes. Membranes were blocked with 5% nonfat dry milk in TBS containing 0.05% Tween 20 (TBST). Primary antibodies were then added to the membranes for incubation, with antibody against ST6Gal-I (a monoclonal generated by the UAB Hybridoma Core Facility), β_1 _integrin (Transduction Laboratories, Lexington, KY), or the V5 epitope (Invitrogen, Carlsbad, CA). Membranes were then washed and incubated with horseradish peroxidase-coupled secondary antibody (Amersham, Piscataway, NJ). The labeled proteins were visualized with enhanced chemiluminescence, and subsequent images were scanned with a Hewlett-Packard Scanjet 5470 c (Wilmington, DE).

### SNA-1 lectin affinity assay

Cell lysates were incubated overnight at 4°C with rotation with 100 μg/mL of the α2–6 sialic acid-specific lectin, SNA-1, conjugated to agarose beads (Vector Laboratories, Burlingame, CA). The lectin-glycoprotein complexes were collected by centrifugation, washed with lysis buffer, and released from the bead complexes by boiling in SDS-PAGE sample buffer. Precipitated proteins were resolved by reducing SDS-PAGE, and immunoblotted to detect β_1 _integrin.

### Stable ST6Gal-I transduction of OV4 cells

An ST6Gal-I cDNA construct, containing a C-terminal V5 tag, was a generous gift from Dr. Karen Colley (University of Illinois, Chicago). This construct was incorporated into a lentiviral vector containing a puromycin-resistance cassette for selection of stably-transduced cells, as previously described [12]. OV4 cells were transduced with the ST6Gal-I lentivirus, and a pooled population of stable clones was obtained by puromycin selection. As a control, OV4 cells were transduced with a lentiviral construct lacking ST6Gal-I ("empty vector" cells). Stable expression of ST6Gal-I was confirmed by immunoblotting for ST6Gal-I, as well as the V5 tag.

### Cell adhesion assay

The parental (P), ST6Gal-I-expressing (ST6), and empty vector-transduced (EV) cells were cultured in serum-free DMEM/F12 media for 48 hours. Cells were disengaged from the culture flasks using CellStripper solution (Cellgro, Herndon, VA) and 8 × 10^4 ^cells were plated onto culture dishes pretreated with 20 μg/mL bovine collagen I and blocked with 2% denatured bovine serum albumin (dBSA). To control for nonspecific binding, cells were also plated onto dishes pretreated with dBSA alone. Cells were allowed to adhere for 30 minutes at 37°C, and then samples were washed gently with PBS. The remaining adherent cells were fixed using formaldehyde and 4% sucrose, and subsequently stained with crystal violet and solubilized with 10% acetic acid. Absorbance of the solution dye was measured at 540 nm.

### Haptotactic collagen I cell migration assay

P, ST6, and EV cells were cultured in serum-free media for 48 hours and disengaged from the culture dishes using CellStripper solution. 2.5 × 10^5^cells were then seeded into the upper wells of Boyden chambers included in the QCM Collagen I Quantitative Cell Migration Assay Kit (Chemicon International). The chambers were lined with 8.0 μm polyethylene terpthalate (PET) membranes coated on the underside with a collagen I concentration gradient. To control for nonspecific migration, cells were also seeded into Boyden chambers with PET membranes coated with BSA. The lower chambers contained 300 μL of conditioned, serum-free NIH3T3 media for the chemoattractant. Cells were allowed to incubate at 37°C for 14 hours, and migration to the underside of the membrane was quantified as per the vendor's staining protocol.

### Cell invasion assay

P, ST6, and EV cells were cultured in serum-free DMEM/F12 media for 48 hours prior to being disengaged from the culture flasks using CellStripper solution. BD BioCoat Growth Factor Reduced (GFR) Matrigel Invasion Chamber (BD Biosciences, San Jose, CA) assay kits were used to measure invasion. 5 × 10^5 ^cells were seeded into the upper wells of Boyden chambers lined with 8.0 μm PET membranes with a thin layer of GFR Matrigel Basement Membrane Matrix. The lower chamber contained 300 μL of conditioned, serum-free NIH3T3 media for the chemoattractant. Cells were incubated at 37°C for 48 hours, and invasion was quantified as per the vendor's staining protocol.

## Results

### A screen of three ovarian carcinoma cell lines reveals differing levels of ST6Gal-I expression

**Figure 1:**
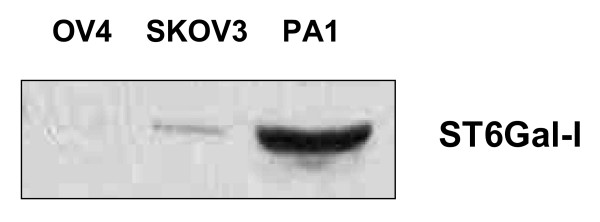
**Screen of three ovarian carcinoma cell lines for ST6Gal-I expression.** PA1, OV4, and SKOV3 cells were grown in culture, lysed, resolved under reducing conditions with SDS-PAGE, and then immunoblotted for ST6Gal-I.

Levels of ST6Gal-I mRNA have been shown to be increased in ovarian carcinoma [19], but, to date, there is no published work characterizing ST6Gal-I protein levels, or its activity *in vitro *or *in vivo*. We chose three established ovarian carcinoma cell lines to screen for the enzyme: PA1, OV4, and SKOV3. To this end, cells were lysed and immunoblotted for ST6Gal-I. As shown in Fig. 1, PA1 demonstrated the highest expression of ST6Gal-I, while OV4 had negligible levels. Expression level in SKOV3 cells was also low relative to PA-1, but significantly higher than in OV-4 cells.

### The level of expression of ST6Gal-I is predictive of β1 integrin hypersialylation

**Figure 2:**
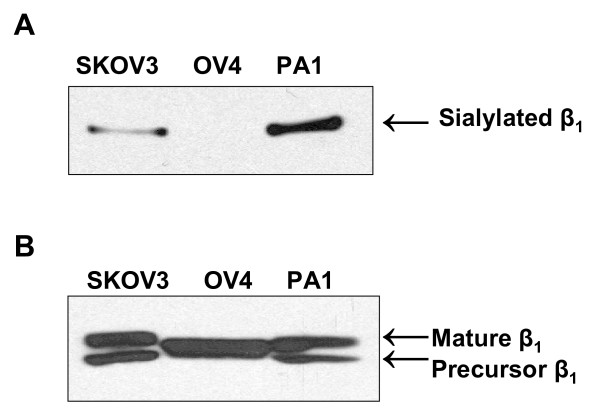
**α2–6 sialylation of β_1 _integrins in three ovarian carcinoma cell lines.*A***, Lysates from PA1, OV4, and SKOV3 cells were incubated with agarose-conjugated SNA, a lectin specific for α2–6 sialic acids. Glycoproteins were precipitated, resolved by SDS-PAGE and immunoblotted for the β_1 _integrin. ***B***, Cell lysates were immunoblotted for the β_1 _integrin to control for total levels of protein expression. The top band in β_1 _immunoblots represents the functional receptor isoform ("mature β_1_"), whereas the bottom band represents a precursor, ER-resident, form of β_1_. Of note, OV4 cells do not appear to express a precursor isoform.

To assess levels of α2–6 sialylation on the ST6Gal-I substrate, β_1_integrin, we evaluated integrin reactivity to SNA-1, a lectin which specifically recognizes α2–6-linked sialic acids. Briefly, cell lysates were incubated with agarose-conjugated SNA-1, and SNA-bound glycoproteins were then collected by centrifugation. The glycoproteins were resolved by SDS-PAGE, and Western blotted for β_1 _integrin (Fig. 2A). In line with the relative amount of ST6Gal-I expression, PA1 had the highest amount of α2–6 sialylation of β_1 _integrin, followed by SKOV3, with OV4 having no detectable α2–6 sialylation of its β_1 _integrin. PA1, SKOV3 and OV4 cell lysates were also immunoblotted for total amounts of β_1 _integrin, which revealed comparable levels of the protein in the three cell lines (Fig. 2B). Interestingly, the higher molecular weight band in β_1 _immunoblots ("mature" isoform, representing the functional receptor) displayed variable electrophoretic mobility for the three cell lines, with the bands from PA1 and SKOV3 cells showing reduced mobility. As we have previously reported, changes in electrophoretic mobility of the mature β_1 _integrin isoform typically reflect variation in the degree of α2–6 sialylation [12,21]. Thus, the increased apparent molecular mass of mature integrins expressed by PA1 and SKOV3 cells is consistent with the observation that these integrins are more heavily sialylated. Of note, the lower band in β_1 _immunoblots is thought to represent a precursor integrin isoform localized to the endoplasmic reticulum, and as such, is not a substrate for ST6Gal-I. The precursor isoform was not observed in OV4 cells.

### Forced expression of ST6Gal-I in OV4

**Figure 3:**
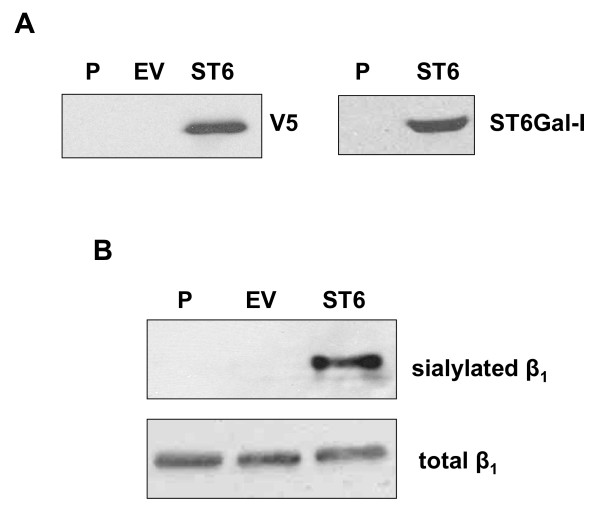
**α2–6 sialylation of β_1 _integrins in ST6Gal-I-expressing OV4 cells.** Parental OV4 cells (P) were stably transduced with a lentiviral vector encoding an ST6Gal-I cDNA fused to a V5 tag (ST6). Cells were also transduced with an empty lentiviral vector as a control (EV). ***A***, Cell lysates were immunoblotted for the V5 tag (left panel) or for ST6Gal-I (right panel) to verify successful transduction of the ST6Gal-I construct. ***B***, Lysates from P, EV, and ST6 cells were SNA-precipitated and immunoblotted for β_1 _integrins to monitor levels of integrin sialylation. Lysates were also immunoblotted for total levels of β_1. _As shown, expression of ST6Gal-I in OV4 cells caused β_1 _integrins to become α2–6 sialylated, verifying that the transduced enzyme was active.

In order to illustrate the role of α2–6 sialylation in modifying integrin-dependent cell behaviors, OV4 cells were stably transduced with a lentiviral vector containing a V5-tagged ST6Gal-I construct (ST6). An empty-vector control cell line (EV) was also generated (note that these cell lines represent a pooled population of stably-transduced clones). Expression of the ST6Gal-I construct was confirmed by Western blotting for both ST6Gal-I and for the V5 tag (Fig. 3A). Neither the parental (P) nor EV cells showed a detectable signal, whereas the ST6 cells showed a strong signal for both ST6Gal-I and the V5 tag.

In order to demonstrate that the ST6Gal-I construct was functionally active, SNA was used to precipitate α2–6 sialylated glycoproteins as described above. The precipitates were then Western blotted for the β_1 _integrin, and, as expected, only the β_1 _integrins from ST6Gal-I expressing cells were found to be α2–6 sialylated (Fig 3B).

### Cells expressing ST6Gal-I show greater adhesion to collagen I

**Figure 4:**
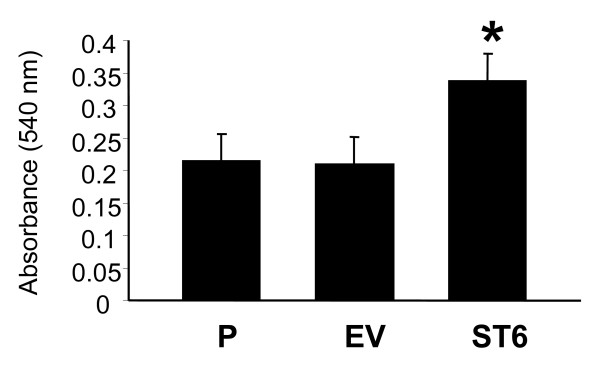
**Cell adhesion to collagen I.** OV4 cells (P, EV, and ST6) were seeded onto culture dishes coated with collagen I, and binding was quantified using a standard crystal violet straining protocol. Data represent means and SEMs of three independent experiments run in triplicate. * denotes P < 0.05, evaluated by ANOVA.

Collagen I is a known β_1 _integrin ligand, and cell attachment to collagen I is integrin-mediated. We have previously reported that α2–6 sialylation of β_1 _integrins enhances the adhesion of colon carcinoma cells to collagen I [12]. Thus, OV4 cells were monitored for binding to collagen I. As shown in Fig. 4, attachment to collagen I was significantly increased in the ST6 cells compared with P (p < 0.01) and EV (p < 0.05) cells. There was no difference in binding to collagen I between P and EV.

### Cells expressing ST6Gal-I show increased haptotactic migration on collagen I

**Figure 5:**
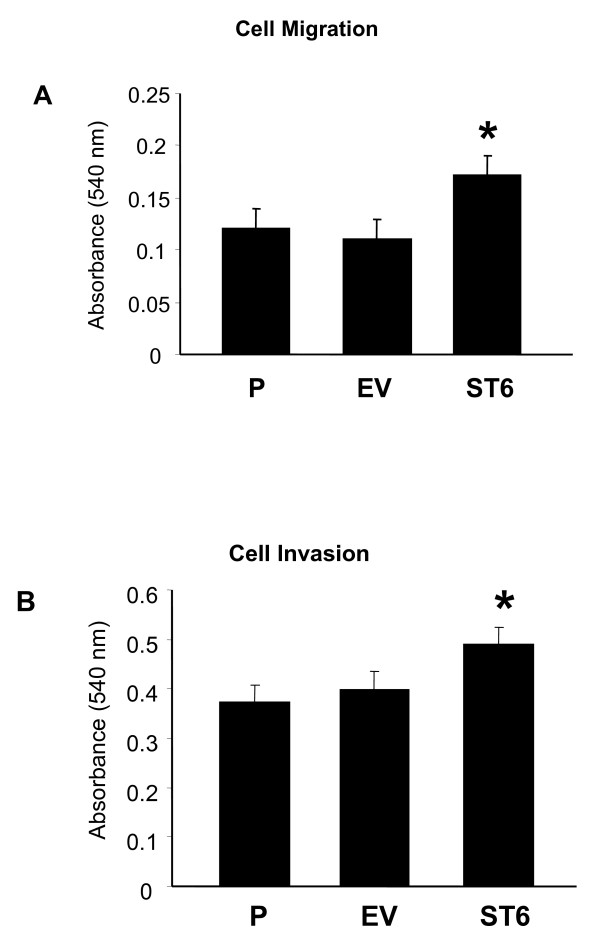
***A***, **Haptotactic migration toward collagen I. P, EV, and ST6 cells were serum starved for 48 hours.** Cells were then seeded in serum-free media into the upper wells of Boyden chambers lined with 8.0 μm PET membranes coated on the underside with a collagen I. The lower chambers contained conditioned NIH3T3 media as a chemoattractant. Cells were allowed to migrate for 14 hours, and cell migration was quantified using the vendor's protocol. ***B***, Invasion of OV4 cells through Matrigel-coated transwells. P, EV, and ST6 cells were serum starved for 48 hours, and then seeded into the upper wells of Boyden chambers lined with Matrigel-coated 8.0 μm PET membranes. The lower chambers contained condition NIH3T3 media as a chemoattractant. Cells were allowed to invade for 48 hours and invasion was quantified using the vendor's protocol. Data represent means and SEMs of three independent experiments run in triplicate. * denotes P < 0.01, evaluated by ANOVA.

A hallmark of advanced ovarian carcinoma is intraperitoneal spread, and therefore cancer cells with a phenotype that includes increased migration might be more apt to metastasize. To evaluate the migratory properties conferred to the OV4 cell line by α2–6 sialylation, we compared the cell lines in a Boyden chamber coated on its underside with a collagen I concentration gradient. Conditioned serum-free NIH 3T3 media was used as a chemoattractant. As shown in Fig. 5A, ST6 cells were more migratory than either P (p < 0.001) or EV (p < 0.001) cells. There was no difference between P and EV migration.

### Cells expressing ST6Gal-I show increased invasion

To determine whether up-regulated ST6Gal-I confers a more invasive phenotype, a cell invasion assay was run. More specifically, cells were applied to the top of a layer of growth-factor reduced Matrigel, coated on the top of a transwell filter. Cells were seeded in serum-free media, with conditioned NIH 3T3 media in the lower chamber as a chemoattractant. Cells were allowed to invade for 48 hours, and the cells migrating through the Matrigel to the underside of the filter were quantified. As shown in Fig. 5B, the ST6 cells were more invasive than either P (p < 0.05), or EV (p < 0.05). No difference was observed in the invasiveness of P and EV cells.

## Discussion

Peritoneal metastasis of epithelial ovarian carcinoma is the primary means of metastatic spread, although a small minority of tumors disseminate via hematogenous or lymphatic routes. At the time of diagnosis, about 70% of patients will have peritoneal spread of the disease, indicative of advanced stage (III-IV), which confers a worse prognosis than if the disease were discovered at an earlier stage. Though the process of peritoneal seeding is poorly understood, the most widely accepted hypothesis is that cells detach from the primary tumor, and are transported via peritoneal fluid throughout the abdomen, eventually attaching themselves to the peritoneal surface. Phenotypically, the tumor cells with the best chance of metastasizing are cells with the ability to escape apoptosis following detachment, while exhibiting increased capacity to adhere to, and invade through the peritoneum, which is exactly the cellular phenotype routinely seen in advanced stage ovarian carcinoma [22]. In the present study, we show that forced expression of ST6Gal-I in ovarian epithelial cells, resulting in α2–6 sialylation of β_1 _integrins, induces increased adhesion and migration on collagen I and invasion through Matrigel. These results suggest that upregulation of ST6Gal-I in ovarian carcinoma may confer a more metastatic phenotype, which mirrors the findings of others' work with colon and breast cancers [13,12].

The regulation of ST6Gal-I expression is multifactorial. Its expression is increased by oncogenic ras [9-11], though a ras mutation is only present in approximately 6% of epithelial ovarian cancers [23]. However, even in the absence of a ras mutation, perturbations in the ras signaling pathway can lead to physiologically activated H-ras, which can be present in as much as 60% of ovarian tumors [24]. Cytokines, such as TNF-α, IL-1, and IL-6, can also induce expression of ST6Gal-I [25,26], and interestingly, IL-1 and IL-6 have been shown to increase ovarian carcinoma cell motility and metastasis, as well as being able to up-regulate TNF-α production [27,28]. Finally, there are data to suggest that steroidal regulation of ST6Gal-I may be of importance in ovarian cancer. Corticosteroids up-regulate α2–6 sialyltransferase activity *in vivo *[29,30], and increase ST6Gal-I mRNA expression *in vitro *[31]. Further, cortisol has been shown to increase invasiveness in the SKOV3 cell line *in vitro *[32]. Estradiol (E_2_) decreases ST6Gal-I expression in a dose dependent fashion in the human breast cancer cell line, MCF-7, an effect reversed with Tamoxifen [33]. A lack of responsiveness to E_2 _in ovarian cancers has been demonstrated in SKOV3 to be due to a mutation in estrogen receptor-α [34], and thus is a plausible explanation for the hypersialylated phenotype despite an estrogenic microenvironment. Based on our findings in the present study, α2–6-hypersialylation may contribute to the invasive phenotype induced by these various modalities by altering the function of the β_1 _integrin receptor.

We have previously shown that ST6Gal-I-mediated sialylation of β_1 _integrins expressed by colon tumor cells increases cell adhesion to, and migration on collagen I [12]. Likewise, α2–6 sialylation of purified integrin receptors enhances receptor binding to collagen I, confirming a critical role for sialylation in regulating integrin function. Collagen I has been shown to be secreted *in vitro *by LP9 mesothelial cells, along with fibronectin, laminin, vitronectin, and collagen types III and IV. *In vivo*, these molecules would contribute to the make up of the extracellular matrix (ECM) that free floating ovarian carcinoma cells would encounter, adhere to, and subsequently invade [35]. β_1 _integrin's importance in the metastasis of ovarian cancer has been repeatedly demonstrated. β_1 _integrin is integral to multicellular spheroid formation [36], adhesion to peritoneal mesothelium [35,37], migration toward a variety of ECM molecules [38], and spheroid disaggregation and invasion [39]. Most studies of altered β_1 _function have focused on either changes in integrin expression or regulation of activity through "inside-out" signaling mechanisms (e.g., conformational changes elicited by the binding of cytosolic molecules to integrin cytoplasmic tails). However, there is growing appreciation for the role of variant sialylation in modulating β_1 _activity.

Given the extensive evidence of hypersialylation in tumor progression, sialyltransferases have been investigated as potential targets for drug therapy [40]. ST6Gal-I acts to catalyze the transfer of the activated sialyl residue from a sugar nucleotide donor to a glycoconjugate acceptor. Strategies designed to halt this process can be aimed at competitively inhibiting the donor with a sugar nucleotide analog, or with an analog of the transition state which binds with many order higher affinity to sialyltransferases than do ground state analogs [41], or by inhibiting the acceptor with a glucoconjugate analog. Another promising avenue of sialyltransferase inhibition is with antisense-oligodeoxynucleotides, which reduce cell surface sialylation without affecting overall cell viability or growth [42]. Challenges remain in developing a sialyltransferase inhibitor that is readily bioavailable, but several strategies to circumvent these problems are under investigation.

## Conclusion

In this study, we have shown that cell behaviors consistent with a metastatic phenotype can be induced in ovarian tumor cells by upregulation of ST6Gal-I, with consequent α2–6 sialylation of β_1 _integrins. Overexpression of ST6Gal-I has previously been implicated in colorectal and breast adenocarcinomas, however, only limited information has been available regarding the role of this enzyme in ovarian cancer. The accumulating evidence indicating that ST6Gal-I-mediated integrin sialylation causes increased cell migration and invasion in multiple tumor types suggests that ST6Gal-I is a promising target for therapeutic intervention.

## Authors' contributions

DRC, FMS and JAL IV were directly involved in data acquisition and analysis. DRC wrote the manuscript with editorial assistance from JAL III and SLB. DRC, SLB and JAL III were responsible for the initial conception and design of the study.
